# A new inexpensive ultrasound-guided central venous catheterization simulation model

**DOI:** 10.1186/s12909-023-04080-z

**Published:** 2023-02-11

**Authors:** Yan Liu, Jiemei Li, Jinzhu Chang, Shaoling Xiao, Wenbo Pei, Lei Wang

**Affiliations:** 1 Department of Anesthesiology, Heping Hospital Affiliated to Changzhi Medical College, Changzhi, 046000 Shanxi China; 2 Clinical Skills Center, Heping Hospital Affiliated to Changzhi Medical College, Changzhi, 046000 Shanxi China; 3 Department of Anesthesiology, Changzhi Medical College, Changzhi, 046000 Shanxi China

**Keywords:** Medical education, Simulation training, Ultrasound model, Central venous catheter (CVC)

## Abstract

**Background:**

Central venous catheters (CVCs) are life-saving tools for fluid therapy during surgery. Ultrasound-guided CVC placement has been shown to be safe and highly efficient. However, it is difficult for medical workers with less experience in ultrasonography to acquire the necessary skill in a short time. Simulation-based training is a good way to enhance the skill of a beginner. Therefore, in this study, we introduced a new, inexpensive and easily implemented model for ultrasound-guided CVC placement training and assessed the feasibility of this model.

**Methods:**

This was a quasi-experimental study. Thirty-three anaesthesiology postgraduate year 2 and 3 residents with strong CVC interest were included in a simulator-based training workshop in a department of anaesthesiology. The simulation model consisted of a piece of pork and two latex catheters filled with red and blue ink. The workshop comprised 3 parts: a 10-min introductory lecture, a 15-min orientation on performing ultrasound-guided CVC insertion based on the model, and a 30-min practice session. Participants completed relevant questionnaires before and after the training. Moreover, an examination was held to evaluate their skill with the novel model.

**Results:**

All participants indicated that the novel model increased their self-perceived confidence in ultrasound-guided catheterization. They also all reported that the model was adequate for training anaesthesiology residents in ultrasound-guided catheterization. A few individuals thought the model did not mimic the progress of CVC insertion (3 of 33). After training, participants did not show a significant difference in the acquisition of central venous catheterization theory. However, their competency with ultrasound-guided CVC placement was enhanced. This was demonstrated not only based on subjective answers to the following questions, namely, “how do you perform central venous catheterization with ultrasound guidance?” (*p* < 0.001), “can you perform ultrasound-guided central venous catheterization?” (*p* < 0.001), and “how much self-confidence do you have in performing ultrasound-guided central venous catheterization?” (*p* < 0.001), but also in objective performance (evaluation of the core step in ultrasound-guided placement (*p* < 0.001)).

**Conclusion:**

The new simulator is a feasible, inexpensive and easily reproducible tool for training anaesthesiologists in ultrasound-guided central venous catheterization. After the simulation-based training workshop, the competency of residents in performing central venous catheterization with ultrasound guidance improved.

## Introduction

Central venous catheters (CVCs) are a common and critical tool used during the perioperative period, and central venous catheterization is carried out in > 5 million individuals each year in the USA [1]. The routine CVC placement method utilizing external anatomical landmarks causes complications in 5–10% of patients [2–4]. Although ultrasound-guided central venous catheterization is safe and efficient [5], the use of this technique is profoundly hindered by a lack of precise visualization and needle tip control for proper positioning [6, 7]. Thus, the effectiveness of this method is influenced by physician experience with ultrasound operation. Beginners may require multiple needle passes, which increases complication rates [8, 9].

Simulation-based medical education (SBME), defined as any educational activity that utilizes simulation aimed to replicate clinical scenarios, is a learner-centred approach that improves medical learner knowledge and performance in a variety of settings [10]. Presently, many simulators are used in simulation-based training, including central venous catheterization [11, 12]. Current training methods rely heavily on commercially available mannequins, which allow trainees to repeatedly and deliberately practice with no risk to patients. However, there are substantial limitations to SBME based on mannequins. The models are generally expensive, with an average cost above $1,500 each [13, 14], which may not be affordable for all clinical institutions. In addition, mannequins must be constantly maintained and regularly replaced for continuous proper functioning. Furthermore, some sonographers have noted the unrealistic haptics associated with mannequins and the different imaging quality between mannequins and human tissues [15].

Animal tissue models are cost-effective and realistic training tools for ultrasound-guided surgeries [16–20]. Pork, as a material for these types of models, is readily available and has multilayered biological tissue; thus, ultrasound images of this tissue are similar to those of human tissue.

Ultrasound-guided CVC placement is an invasive operation, and we developed a novel, feasible model based on pork that could be rapidly built and easily replicated for simulation training before performing surgeries on actual patients. We aimed to assess the implementation, acceptability, and practicality of the model. We also researched whether this model is a feasible training tool for beginners learning how to perform ultrasound-guided CVC placement.

## Methods

This quasi-experimental study included a pretest and posttest, comparing responses from participants who received simulation-based training sessions because of limited resources [21]. The study was performed at the Department of Anaesthesiology, Heping Hospital affiliated with Changzhi Medical College of China, from December 2020 to January 2022. This work was carried out in accordance with the Declaration of Helsinki (2000) of the World Medical Association. The Medical Ethics Committee of Changzhi Medical College determined that this trial was exempt from ethics committee review.

### Participants and sample size

We assumed that participants would have a general understanding of ultrasound-guided CVC placement, which means that 50% of questionnaires were correctly completed in this study, corresponding to a score of at least 4/8 points. To detect a 10% improvement in score, with 1% significance and 80% power, a sample of 20 students was needed.

Because postgraduate year 1 students had not undergone training regarding preparation for CVC placement procedures, such as washing hands, wearing gloves, and organizing equipment, they were excluded from this study. All anaesthesiology residents in postgraduate years 2 and 3 who agreed to participate in the study were eligible for enrolment.

### Model building

**Fig. 1:**
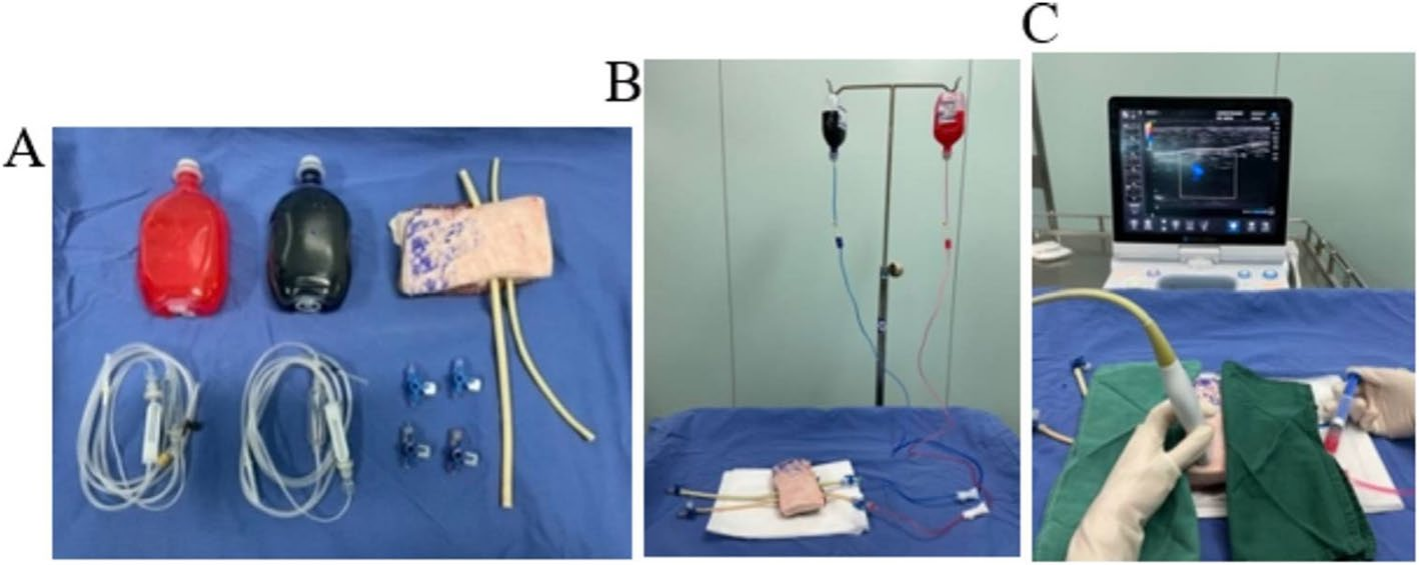
Images of the tissue model. **A** CVC model’s construction materials. **B** Overall structure of the model. **C** In short-axis view, researcher is utilizing color doppler while squeezing one of the latex catheters with a syringe to simulate arterial flow

The CVC model was built immediately prior to simulation from diverse readily available materials routinely found in the operating room. The materials needed for model assembly are shown in Table 1 and Fig. 1A.

**Table 1:** Model Building Materials and Costs

Material	Amount	Unit Cost, US$	Total Cost, US$
Pork	1 (16 cm*10 cm)	3.5	3.5
Latex catheter	2 (16 Fr and 22 Fr)	0.45	0.9
Red and blue ink	each bottle of	0.15	0.3
Infusion	2	0.13	0.26
Stopcock	4	0.35	1.4
0.9% sodium chloride brine(250 ml)	2	0.16	0.32
Total material cost			6.68

**Fig. 2:**
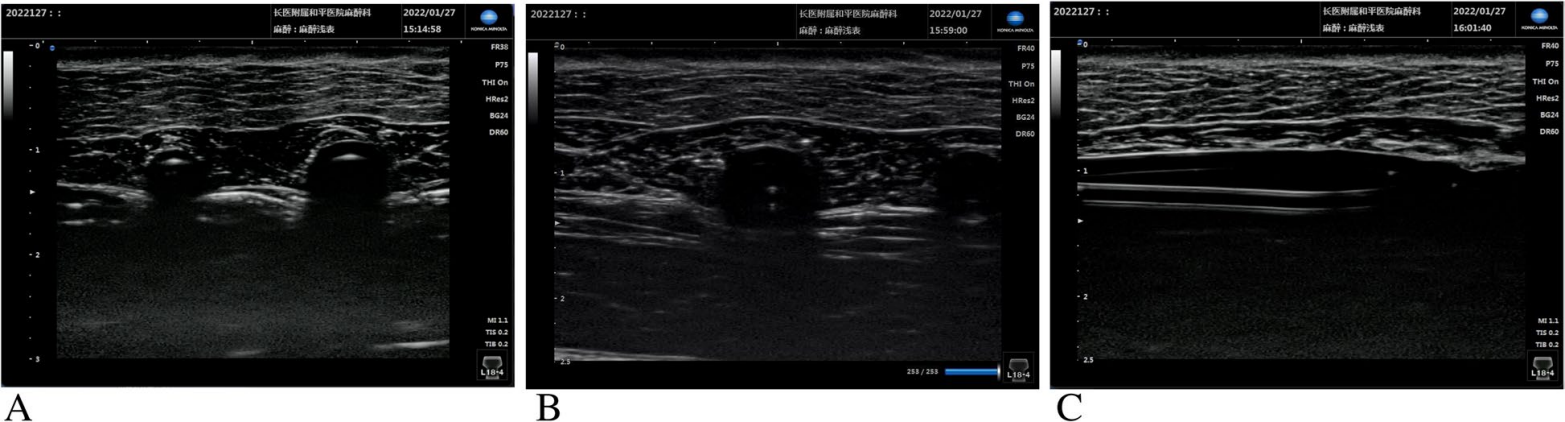
Vascular ultrasound images. when researcher is utilizing a high-frequency ultrasound probe scan the model. **A** Vessels (left for artery and right for vein). **B** Confirmation of catheter’s position in the short axis view (sound beam was perpendicular to course of the vessels). **C** Confirmation of catheter’s position in the long axis view (sound beam paralleled to course of the vessels)

The model mainly consisted of a piece of pork and two latex catheters filled with coloured water (blue for the vein and red for the artery) (Fig. 1B). First, two tunnels were pierced through the pork, and latex catheters were inserted into them. Second, the infusion device and the proximal ends of blood vessels were connected, with the distal ends closed with stopcocks. Then, coloured water was added. Residents performed ultrasound on the model, which simulates the right internal jugular vein (IJV) of humans (artery on the left and vein on the right) (Fig. 1C). The images of two vessels could be seen under ultrasound (left for artery, right for vein) (Fig. 2A).

### Ultrasound-Guided CVC

Since the internal jugular vein (IJV) is relatively superficial, a high-frequency ultrasound probe (7–12 Hz, SONIMAGE HS1) yields clear images. When the probe-emitted sound beam was perpendicular to the course of the vessels, the vein could be visualized in the short axis view. In the short-axis view, the vein diameter as well as the location of the surrounding artery was displayed clearly. Then, by adjusting the probe orientation, making the sound beam parallel to the course of the vessels, the vein could be visualized in the long-axis view. Ultrasound probes are most preferably utilized by starting with the short-axis view, confirming needle insertion in the vein, and utilizing the long-axis view after appropriate needle insertion improves visualization and needle path monitoring [22]. The various ultrasound views are depicted in Fig. 2 (B, C). Then, catheterization was carried out based on standard procedures.

### Simulator-based training

**Fig. 3:**
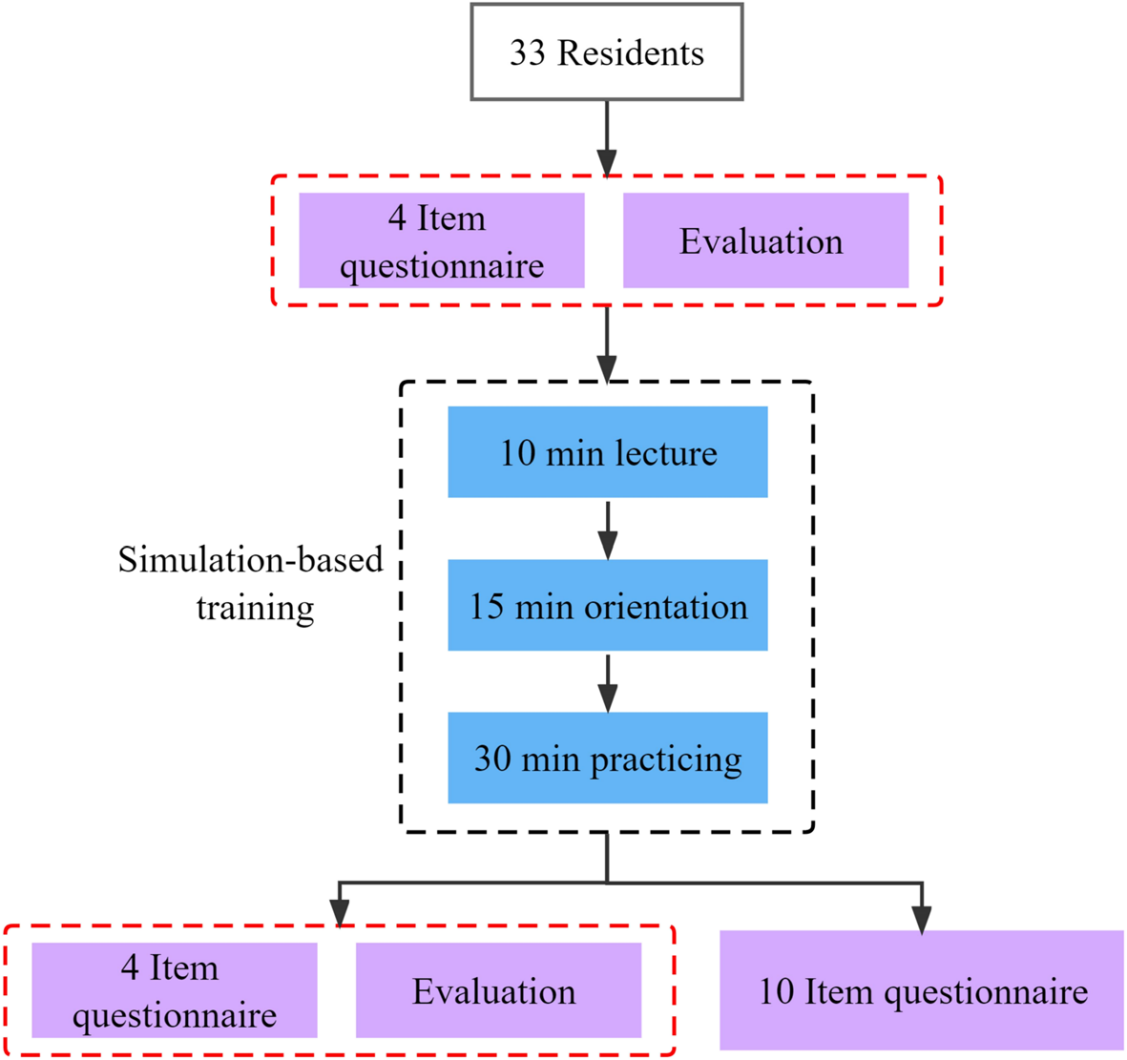
Study procedure

The training comprised 3 parts: 1) a 10-min introductory lecture, 2) a 15-min orientation on performing ultrasound-guided CVC insertion based on the model, and 3) a 30-min practice session. After participants received the pretest questionnaire and completed the first examination about the core steps of ultrasound-guided central venous catheterization, the introductory lecture was given about the theories of central venous catheterization and ultrasound, which were designed to arm participants with conceptual knowledge prior to performing ultrasound-guided catheterization. Then, the 15-min orientation/demonstration on performing central venous catheterization based on the model was presented by an experienced ultrasound fellow to enhance the knowledge acquired by participants in the previous part of the training. Finally, to ensure that everyone could obtain equal practice opportunities, all participants were assigned to 3 groups to practice US-guided CVC placement using the model. The workshop director and fellows provided individual small-group instructions and guidance on the technique of ultrasound-guided CVC placement on the simulator. Each participant practised for 30 min. All participants attended a single hands-on session and received the posttest questionnaires and completed the second examination after training. The whole training process is shown in Fig. 3.

### Pretest/Posttest questionnaires and examinations

The 4-item questionnaire (Table 2) and the evaluation of the core steps of ultrasound-guided central venous catheterization (Table 3) were both obtained before the introductory didactic lecture and after the workshop. The design of Table 2 referenced several similar studies with the same purpose, which aimed to evaluate the acquisition of ultrasound-guided CVC placement skill depending on participant self-assessment about the theory of central venous catheterization, the use of ultrasound, their confidence in ultrasound-guided CVC placement, etc. Table 3 was designed to objectively assess the participants’ skill. Evaluation criteria were established according to the European Society of Anaesthesiology guidelines on perioperative use of ultrasound guidance for vascular access [23]. By analysing the results of Tables 2 and  3, the researchers aimed to determine the effectiveness of this simulator-based training.

**Table 2:** The 4-item Questionnaire

Question		Completely Unfamiliar	Unfamiliar	General	Familiar	Completely Familiar
I	Have you understanded the theory of central venous catheterization?	1	2	3	4	5
II	Do you know how to perform central venous catheterization with ultrasound?	1	2	3	4	5
III	Can you operate ultrasound-guided central venous catheterization now?	1	2	3	4	5
IV	How much self-confidence in ultrasound-guided central venous catheterization?	1	2	3	4	5

**Table 3:** Evaluation table of the core steps of ultrasound-guided central venous catheterization

Content	Full marks
Ultrasound-guided location	1
Ultrasound-guided short-axis planar approach	1
Pumping back while feeding the needle until the blood is returned smoothly	1
Determining whether a vessel is a central vein after penetration	1
Placement of a guidewire (about 15 cm)	1
Expansion of the skin with needle	1
The catheter is placed about 12 cm with saline syringe back to pump through	1
Suture or fixation of catheters, dressing coverage	1
Total Scores	8

The 10-item postworkshop questionnaire (Table 4) obtained following CVC simulation utilized a 5-point Likert scale (1 [strongly disagree] to 5 [strongly agree]). The responses “agree” and “strongly agree” were considered general agreement, while “disagree” and “strongly disagree” denoted general disagreement. Questions in this table were used to assess the feasibility of the novel model from its implementation, acceptability, and practicality.

**Table 4:** The 10-item postworkshop Questionnaire

Question		Strongly Disagree	Disagree	Neither Agree nor Disagree	Agree	Strongly Agree
Fidelity						
1	This model is similar to human anatomy	1	2	3	4	5
2	This model has realistic feeling like human tissue	1	2	3	4	5
3	This model provided clear ultrasound image of vascular structures and needle	1	2	3	4	5
4	This model mimics central venous catheter the progress of insertion	1	2	3	4	5
Convenience						
5	The CVC model is easy to use	1	2	3	4	5
6	I prefer this model to existing CVC models	1	2	3	4	5
Competency						
7	This model is adequate for training anesthesiology residents	1	2	3	4	5
8	This model would increase competency in central venous catheterization	1	2	3	4	5
9	This model would increase confidence in central venous catheterization	1	2	3	4	5
10	The necessity of this training	1	2	3	4	5

Participants included in this study received a test (including a questionnaire and an examination) that was completed individually without intervention from other participants. The participants were blinded to the survey’s purpose, and the test score did not influence their residential standard training.

### Statistics and analysis

The age and sex of the residents were recorded. To evaluate the cost of the model, researchers kept a record of construction time and expense and calculated an average. Points of every questionnaire and evaluation of the core steps of ultrasound-guided central venous catheterization were also analysed. To protect the privacy of participants, researchers were unable to identify the questionnaire owner, and only univariate descriptive statistics were obtained.

Data analyses were carried out with SPSS 26.0 (IBM Corporation, USA). Data normality was assessed by the Kolmogorov‒Smirnov test. Continuous data are presented as the mean ± standard deviation (SD). Pretest and posttest comparisons were made by paired t test for continuous data. A two-sided *p* < 0.001 indicated statistical significance.

## Results

A total of 33 residents participated in this study, namely, 18 postgraduate year 3 residents and 15 postgraduate year 2 residents. The mean age of the residents was 27.61 ± 0.25 years (median, 28 years; range, 26–32 years). There were 13 men and 20 women. The residents had no prior experience in ultrasound-guided CVC placement.

According to the results of the 4-item questionnaire (Table 2), after training, participants showed differences in their competency in using ultrasound to help with central venous catheterization. For the questions “Do you know how to perform central venous catheterization with ultrasound?”, “Can you perform ultrasound-guided central venous catheterization now?” and “How much self-confidence do you have in performing ultrasound-guided central venous catheterization?”, the difference was significant (*p* < 0.001; Table 5). Additionally, evaluation of their skill improved, and scores were markedly different (5.79 ± 0.78 vs. 7.64 ± 0.49, *p* < 0.001; Table 5). However, for question I, “Do you understand central venous catheterization theory?”, the difference did not reach statistical significance (4.09 ± 0.46 vs. 4.18 ± 0.39, *p* = 0.083). This finding was likely influenced by the fact that postgraduate year 2 and 3 residents have a course that discusses central venous catheterization theory.

**Table 5:** Comparison between the 4-item questionnaire and CVC scores (M ± SD)

	Questions				CVC
	Q I	Q II	Q III	Q IV	
Pre-workshop	4.09 ± 0.46	3.03 ± 0.53	3.64 ± 0.49	3.73 ± 0.45	5.79 ± 0.78
Post-workshop	4.18 ± 0.39	4.03 ± 0.39	4.79 ± 0.42	4.88 ± 0.33	7.64 ± 0.49
t	1.789	16.248	18.167	18.167	20.923
p	0.083	< 0.001	< 0.001	< 0.001	< 0.001

**Fig. 4:**
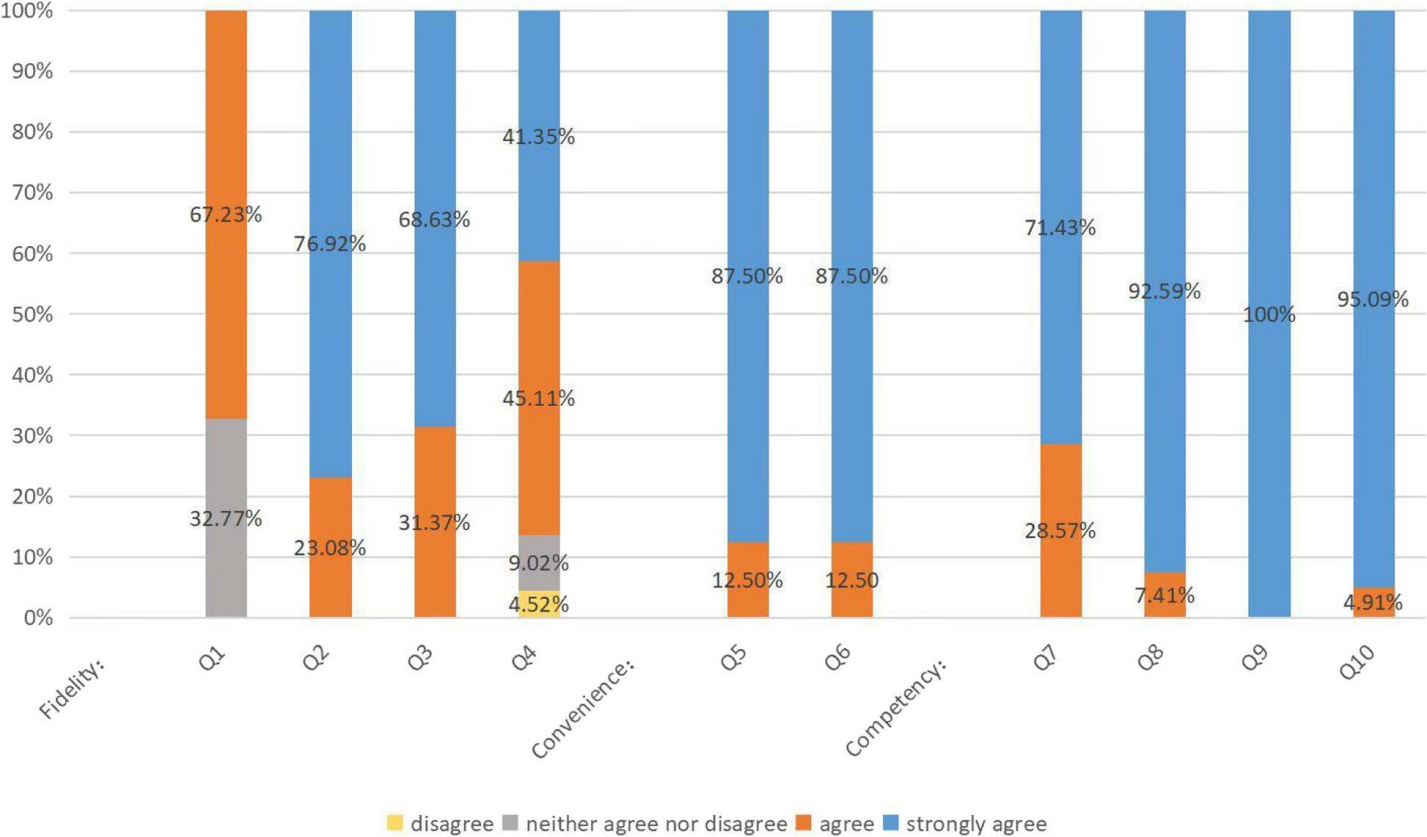
Post-questionnaire responses showing distribution frequencies for the surveyed questions

In total, 6 models were built in this training. The mean construction time required to build a model was 7.3 min. The model cost US$ 6.68 on average, with an overall cost of US$ 40.08. Materials and costs are shown in Table 1. The frequency of distribution of all responses for the 10-item questionnaire is shown in Fig. 4. Significantly, all participants indicated that the model completely increased their self-perceived confidence in performing ultrasound-guided CVC placement and that they thought the model could help appropriately teach CVC placement to anaesthesiology residents. Additionally, 33 participants thought the model was “easy to use” and were highly satisfied with the model development of tissue feeling and accuracy in terms of needle positioning. However, a few participants indicated that this model did not mimic the progress of CVC insertion (3/33 [4.52%]).

## Discussion

Using materials readily available in the operating room, we developed a novel, feasible ultrasound-guided CVC model that could be rapidly built and easily replicated. After training, postgraduate year 2 and 3 residents exhibited enhanced knowledge and competency regarding ultrasound-guided CVC placement [24].

How to get the junior physician to acquire invasive procedures in a short time is a critical issue in medical education. The effectiveness of simulation-based learning has been reported in studies [11, 25, 26]. Numerous vascular training models have been designed for training. For example, one study used a mixed reality trainer, which can project a 3D virtual anatomical image of needle placement tracing, to promote interactivity, 95.2% of 62 participants agreed that visual ability was helpful in placement and 93.5% participants thought that mixed reality should be included in the training [27]. In another study, the researchers build a simulation model that can automatically reproduce the blood flow of vessels and help residents distinuish arteries and veins in ultrasonography [28]. A total of 11 residents significantly enhanced their knowledge and confidence regarding the discrimination of arteries and veins [28]. Students improved manipulation ability through training on these technological or home-made modes.

One of the benefits of simulation-based training highlighted is better comprehension, which can be partially attributed to decreased levels of anxiety among learners [29]. Stress was reduced during the training, and most of that stress was caused by patient bias. An earlier procedural training leads to more productive residents [30]. Students’ abilities were assessed before the training, and educators pay attention to the lack of students’ abilities on a continuous basis. This kind of competency-based education could promote quality of health care and result in patient-relevant outcome [31].

## Limitations

The proposed model had many limitations. First, it used fresh pork belly, which can simulate more tissue feel and realistic ultrasound quality compared with human tissues; however, muscle contraction after freezing could affect image quality. Therefore, each model can only be used for a single day. Second, latex catheters were used to simulate blood vessels, which are different from the actual anatomy. They are more flexible than real blood vessels during puncture and can reduce puncture-related exosmosis compared with other material catheters. Finally, although the ease of latex catheter replacement minimizes degradation, pork tissue still loses its capacity after repeated punctures. There were also several methodological limitations in this study. Because of limited sources, such as a small number of residents, we did not design a randomly controlled study. Additionally, ultrasound-guided CVC placement is an invasive operation, and any further verification of this study result on actual patients without permission was forbidden by our institutional review board exemption.

## Conclusions

Despite the limitations, our new ultrasound-guided CVC placement model provides a feasible, inexpensive, easily reproducible tool for training anaesthesiologists in ultrasound-guided CVC placement. In addition, it is inexpensive, which allows potentially broad application of simulation-based training in all hospitals, especially resource-limited institutions.

## Data Availability

The datasets used and/or analysed during the current study are available from the corresponding author on reasonable request.

## Abbreviations

CVC: Central venous catheter
IJV: Internal jugular vein
SBME: Simulation-based medical education
